# Knowledge Helps: Handling Rare Diseases in Regular Schools

**DOI:** 10.5334/cie.99

**Published:** 2024-02-15

**Authors:** Nicola Sommer, Julia Klug

**Affiliations:** 1 University of Education in Salzburg, AT

**Keywords:** inclusion, rare diseases, school, teaching unit, online tool

## Abstract

Thanks to medical successes and new treatment options, children and adolescents with rare diseases can now attend school more often playing an important role in their recovery as well as improving their social inclusion. For this reason, it is important for teachers to address the issue and acquire skills in dealing with rare diseases. In this context, a multi-professional team at the Salzburg University of Teacher Education organized a blended learning seminar on the topic of rare diseases at schools. Participants were provided with videos, texts, and case studies on a learning platform, which were worked on over a period of three weeks. There were also two online lectures in which questions could be asked. In order to evaluate the tool, 21 participants took part in a quantitative longitudinal study by means of a pretest and a posttest with a four-month interval. The participants completed a questionnaire consisting of a competence screening dealing with rare diseases together with questions to measure general and teacher self-efficacy.

As expected, there was a statistically significant increase in both general and teacher self-efficacy with medium effect sizes. In addition, the theoretical and practical skills for supporting affected students at school were also shown to improve in a self-assessment. In view of the positive response from participants, it is recommended to further expand this offering in order to reach a broader population of teachers.

In other words, only through raising awareness and increasing the competence of professionals working in schools can an environment be created for affected children and adolescents in which their specific needs are addressed.

## Rare Diseases

A disease is considered rare if it affects less than 5 per 10,000 people in the European Union (EU). Worldwide, 3.5 to 5.9% of the population are currently affected by rare diseases which is estimated to be about 18,000,000 to 30,000,000 people in the EU (European Commission 2023). This means that between 3,600,000 and 6,000,000 children and young people up to the age of 19 are affected in the EU.

Of the approximately 6,000 rare diseases identified, 72% are caused by genetic modifications, while others can be caused by allergies, environmental causes, or various infections. About 70% of rare diseases emerge in childhood (Nguengang Wakap et al. 2020).

Despite many differences, some common characteristics of rare diseases can be identified: rare diseases are usually severe, chronic, often degenerative and life-threatening, with a tendency to manifest in childhood. As a consequence, the quality of life for individuals with rare diseases is significantly impaired due to a loss of autonomy; they and their relatives experience a high psychosocial burden because of the scarcity of therapeutic measures and practical support for daily life. Moreover, many of these diseases cannot be cured because there are no effective therapies and, in many cases only symptomatic treatments are available to improve life expectancy and quality of life (Anderson, Elliott & Zurynski 2013; EURORDIS 2005).

In fact, rare diseases are numerous and vastly heterogeneous – thus inevitably, only a few are preventable or curable and most have a chronic course with many leading to premature death. At least, low prevalence, lack of knowledge about the disease, and the scarcely available treatments, have led to the creation of various initiatives (Göbel & Gortner 2011). For example, Orphanet (2020) is a network of 40 countries in Europe and other countries around the globe that aims to improve knowledge about rare diseases. This includes initiatives such as enhancing the speed and accuracy of diagnosis as well as providing competent care and treatment for affected individuals (Kinderliga 2022). The average time from discovery to diagnosis is three years (Pro Rare Austria 2021). Therefore, expedited diagnosis is crucial to prevent diverse psychosocial impacts on the entire family and its surroundings (Kinderliga 2022).

Action plans for rare diseases have been developed in many member countries of Orphanet. Subsequently, this led to recommendations of measures to improve the health of people with rare diseases. That is, the measures taken in many European countries have already led to improvements in the lives of people with rare diseases. In order to take this further, a coordinated approach by all stakeholders is required (Eidt et al. 2009).

The Eurobarometer survey on rare diseases (DG SANTE 2011) concluded that Europeans generally know what rare diseases are, but detailed knowledge and awareness remain low. The level of familiarity with specific cases of rare diseases varies widely. While conditions such as cystic fibrosis or hemophilia are well known, knowledge of other conditions is lacking. Here, the public generally expresses strong support for initiatives related to rare diseases at both the national and international level.

In this respect, the study by Voigtländer et al. (2012) surveyed those affected as well as stakeholders on the situation of people suffering from rare diseases. Results show that a clear majority of respondents perceive an increased awareness of rare diseases and an improved level of general and detailed knowledge. To further expand this positive development, targeted public relations with the help of educational outreach and training should be continued (Voigtländer et al. 2012). Additional activities related to rare diseases in Europe are reported by the European Union Committee of Experts on Rare Diseases (EUCERD) (Aymé & Rodwell 2012).

## School and Rare Diseases

In recent decades, a shift from previously threatening infectious childhood diseases to chronic and rare diseases have been observed. Medical successes have led to an increase in life expectancy and quality of life allowing many of those affected by rare diseases to participate in regular education. Nevertheless, there are some hurdles for these children in everyday school life confirming that why teachers or student teachers should be well informed and trained (Voigtländer et al. 2012).

On the one hand, Pediatric care is very complex and requires interdisciplinary as well as transdisciplinary approaches. On the other hand, the different environments in which children live must be considered. Thus, school plays an important role in this educational and social context (Rosselló et al. 2018). International guidelines recommend increased awareness in schools and support for educators to meet these requirements. However, at present there is still a lack of easily understandable and didactically well-prepared information that facilitates and enriches everyday school life for all those involved. There is a call for the development of practical guidelines for educational institutions that help raise awareness of children with rare diseases, improve quality of life for families by simplifying procedures in the educational sector and increase participation of children with rare diseases in school (Rosselló et al. 2018).

Indeed, the inclusion of children and adolescents with rare diseases in schools poses challenges to educational institutions, teachers and other professionals working in schools. A study by Verger et al. (2020) aimed to further examine these challenges. The study determined ten categories that influence a healthy, inclusive, and equitable educational experience for students with rare diseases: diagnosis, official recognition, belonging, absences, coordination, curriculum adaptations, homework, autonomy, staff resources and peer support. Clearly, providing all these components is a complex process influenced by the child’s particular health status, the family’s level of empowerment, the availability of resources, and the commitment of the school or health care providers (Verger et al. 2020).

Teachers who have already had previous experience with both inclusive programs and teaching have a more positive attitude concerning the inclusion of children and adolescents. Teacher training also plays an important role in the development of positive attitudes (Avramidis, Bayliss & Burden 2000). For instance, in Austria student teachers are already presented during their basic training with information about theoretical and legal foundation of inclusion and deal with attitudes towards disability. In addition, students can choose inclusive education as specialization as part of their studies (Holzinger et al. 2019). The limits of inclusion are defined less by the individual needs of children and adolescents with illnesses or disabilities, but rather by the specific conditions in schools (Gasterstädt & Urban 2016).

The concerns of those affected and their families regarding their own anxieties about the future, the fear of not being taken seriously or accepted, stigmatization, unclear perspectives, and dependencies must be considered (Pro Rare Austria 2019). Therefore, it is important that teachers are informed and sensitized about this issue knowing how to handle it in school.

## The Present Study

### Training Course Content

The aim of the course was to educate teachers about rare diseases, raise awareness about the topic, and demonstrate the pedagogical impact of promoting a positive approach to diversity. For this purpose, an online seminar was developed by the Salzburg University of Education in collaboration with the Austrian Society for Pediatric and Adolescent Medicine (ÖGKJ) and Pro Rare Austria, based on a learning platform. The seminar was accompanied by two introductory online lectures with included consultation hours.

The seminar was divided into two modules. In the first module, the basics of rare diseases and the medical perspective on the topic were conveyed. In the second module, participants learned about the potential impact of rare diseases on daily school life and became familiar with various medical conditions from both a medical and a patient’s perspective. To facilitate the transfer of theoretical input into practice, each module was accompanied by case studies. In addition, participants were challenged to develop a lesson plan on the topic of rare diseases. The best lesson plans were honored in a competition organized by Eurordis (European Organization for Rare Diseases) in connection to the global rare disease day campaign (Link to the winning project: https://www.rarediseaseday.org/downloads/school-toolkit-for-children-7-to-8-year-olds/). Participants received a certificate upon successful completion of the course.

### Objective of the Evaluation

The goal of the evaluation was to examine if the self-efficacy and self-assessment of competence in working with a child affected by a rare disease, among current and future teachers, rise as a result of participating in the online seminar. Additionally, participants’ prior knowledge and experience with the topic were assessed and a retrospective evaluation of competence acquisition was conducted. No other classes have been developed for current and future teachers so far underscoring the significance of this study. It is crucial to raise awareness among professionals working in schools and provide them with opportunities for further education. This could increase self-efficacy in working with affected individuals and build both theoretical and practical skills.

## Method

### Design

The study took place at the University of Education Salzburg, which provides teacher training and professional development. A course on dealing with rare diseases in schools was offered online to reach teachers and teacher education students throughout Austria in a low-threshold manner. A pretest and a posttest design were applied to identify changes in participants’ self-efficacy and self-assessed competencies in dealing with rare diseases in schools after the course compared to a baseline before the course.

### Participants and Procedure

The participants of the Winter 2022 semester online seminar (Knowledge Helps: Handling Rare Diseases in Regular Schools) involved 27 students and 24 teachers. In the beginning and at the end of the seminar participants were sent an online questionnaire, 51 participants undertook the pretest and 26 participants completed the posttest. Furthermore, 21 pretests together with 21 posttests were analyzed. Of these, 18 were female (86%), 43% were between 20 and 29 years old, 5% between 30 and 39 years, 29% between 40 and 49 years, 19% between 50 and 59 years, and 5% 60 years or older. 48% were teachers and student teachers, and one person indicated educational counseling as their profession. 71% of the participants work in primary education, 10% in secondary education. Further, the rest work at a different level or did not fill in this section. Teachers and students from all Austrian federal states participated in the survey, with the largest proportions coming from Salzburg (33%), Upper Austria (29%), and Vienna (19%). Participation in the online data collection was voluntary and anonymous. The study was approved by the rectorate of the University of Education Salzburg in Austria.

### Instruments

This quantitative longitudinal study consisted of two measurement points: the first at the beginning of the online seminar (pretest) and the second at the end (posttest). In addition to demographic data, information about prior knowledge and experience with rare diseases was collected. Prior knowledge about rare diseases was assessed with six items (e.g., Did you have any prior knowledge about the needs of children and adolescents with rare diseases before the online seminar?) on a Likert scale ranging from 1 (no knowledge at all) to 6 (a lot of knowledge). The six items related to prior knowledge formed a scale with an internal consistency of α = .94. Previous experiences were measured with the question: Did you already have personal experience in caring for students with rare diseases before the online seminar? This was also rated on a Likert scale from 1 (no prior experience) to 6 (a lot of prior experience).

To assess self-perceived competencies in dealing with rare diseases, a competency screening questionnaire according to Bergsmann et al. (2017; CSQ-HE) was used. Ten competencies for dealing with rare diseases, previously compiled in a competency model by Gutzweiler, Neese, and In-Albon (2020), were inserted into this flexible framework for competency assessment in various areas (e.g., handling acute complications; organizing and conducting special teaching settings considering the disease). Participants then self-rated their knowledge (cognitive aspect) and their ability to apply it (practical aspect) on competency levels from 0 (no knowledge/no practice) to 6 (creation) for each of these competencies. The internal consistency of the scales for both aspects was determined as α = .98.

General self-efficacy expectations were assessed with three items using the ASKU (Beierlein et al. 2012) and achieved an internal consistency of α = .84. Teacher self-efficacy was measured with the WirkLehr scale (Schwarzer & Schmitz 2002) (10 items, α = .88 in the present sample). For the evaluation of reactions to the online seminar (Kirkpatrick & Kirkpatrick 2006), participants were asked for a general assessment of the course (e.g., valuable for work in school). These were answered on a six-point Likert scale from 1 (strongly disagree) to 6 (strongly agree). Participants were also asked about the organization of the course (e.g., information, goals, online mode). These questions were answered on a four-point scale from not at all (4), a little (3), mostly (2), to completely (1).

### Analyses

All quantitative analyses were conducted using SPSS 28. Reliability analyses and descriptive analyses were computed for all scales. Since the data from the competency screening questionnaire are not interval-scaled but ordinal-scaled, Wilcoxon tests for related samples – as a non-parametric analysis method – were employed to determine a change in competency levels from pretest to posttest. To test for a change in (teacher) self-efficacy from pretest to posttest, paired-sample t-tests were utilized.

## Results

### Prior Knowledge and Experience in Handling Rare Diseases

Participants displayed a relatively low mean prior knowledge with M = 2.24 (SD = .95, Min = 1, Max = 4.5) on a scale from 1 (*no prior knowledge at all*) to 6 (*a lot of prior knowledge*). A similar picture was found for prior experience with M = 1.9 (SD = 1.3, Min = 1, Max = 5) on a scale of 1 (*no prior experience at all*) to 6 (*a lot of prior experience*).

### Change in Self-assessed Competencies in Handling Rare Diseases

**Figure 1:**
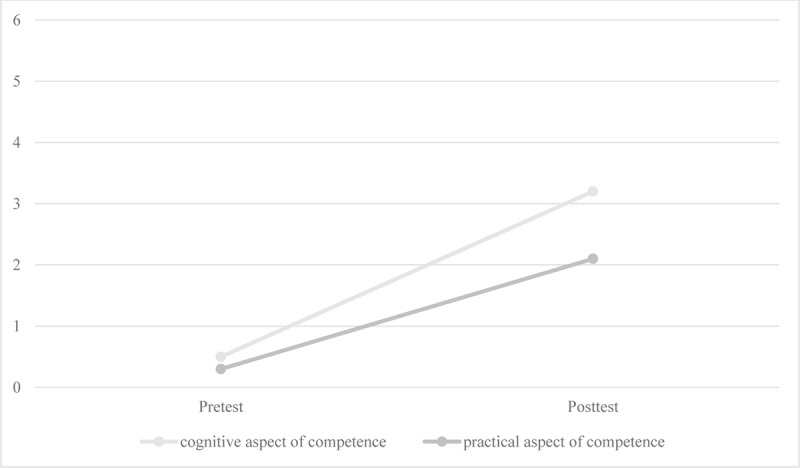
Self-assessed competencies pre- and post-intervention.

To test whether self-assessed competencies in handling rare diseases significantly changed from pretest to posttest, Wilcoxon tests for connected samples were performed. The assessment of both cognitive and practical aspects of rare disease management competencies increased significantly from pretest to posttest (n = 21, z_cognitive_ = 3.70, p < .001; z_practical_ = 3.36, p < .001). Figure 1 illustrates the increase in median self-assessed levels of competency in handling rare diseases from pretest to posttest. Participants started between level 0 (*no knowledge/no exercise*) and level 1 (*threshold; one knows single unrelated facts that one can recognize or one can perform single simple clearly guided activities*) and reached level 3 in the cognitive aspect (*interconnection; one knows many facts and can connect them; one can reason and draw conclusions*) and level 2 in the practical aspect (*foundation; one can perform a variety of clearly guided activities and work according to a guideline*) after participation. In paired-sample t-tests, there was a statistically significant increase from pretest to posttest with a medium effect size (dASKU = .67; dWirkLehr = .40) for both general self-efficacy expectations (t = –2.39, df = 20, p_one-tailed_ < .05) and teacher self-efficacy (t = –2.00, df = 20, p_one-tailed_ < .05). Table 1 shows the descriptive statistics for both forms of self-efficacy in pretest and posttest.

**Table 1:** Descriptive statistics for participants’ (teacher) self-efficacy in pretest and posttest.


	M	SD	MIN	MAX

Self-efficacy pretest	2.84	.64	1.33	4.00

Self-efficacy posttest	3.19	.57	2.00	4.00

Teacher self-efficacy pretest	3.05	.51	1.90	3.80

Teacher self-efficacy posttest	3.22	.39	2.60	3.90


### Competition for Ideas

The best six entries of the competition for ideas for a teaching unit on rare diseases were selected by a jury of Pro Rare Austria and submitted to Eurordis. The selected examples were reviewed by an international expert panel of educators familiar with rare diseases, and their feedback was shared with the participants. One teaching example was selected due to its ease of implementation in any classroom around the world.

### Evaluation of the Course by the Participants

**Figure 2:**
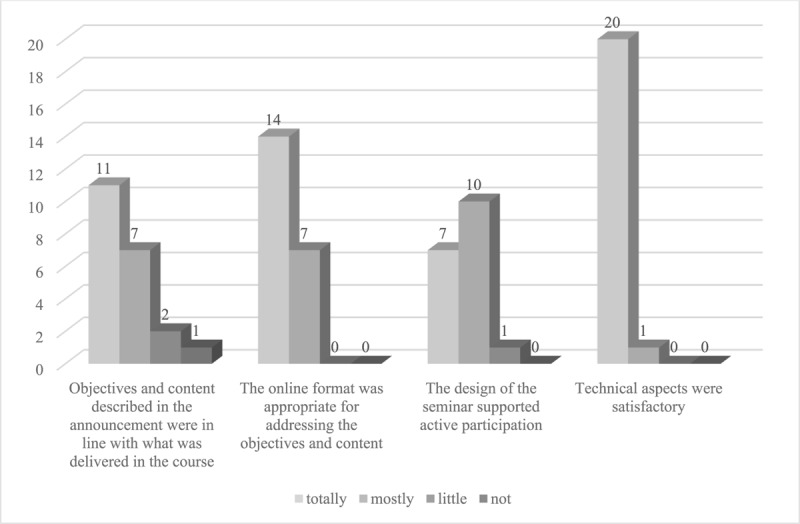
Course evaluation frequencies for organizational aspects.

**Figure 3:**
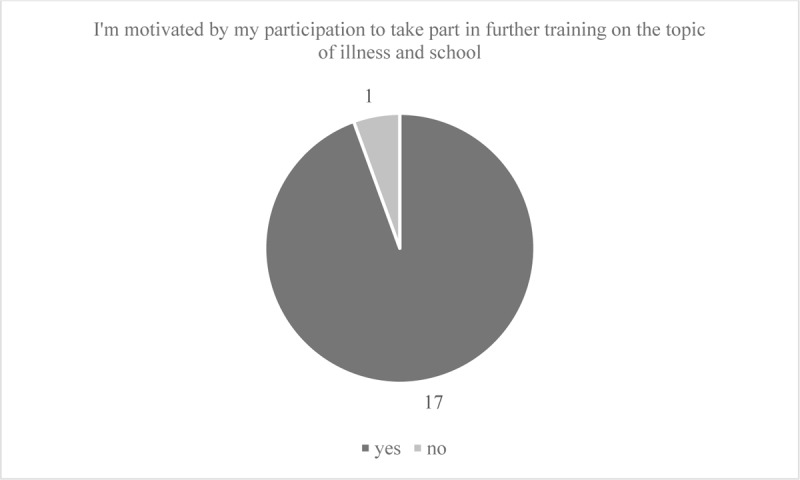
Motivation to participate in further training on the topic of illness and school.

On average, participants enjoyed attending the seminar. The participants also think that they benefited from the seminar for their work at school and feel well prepared for future work with students with rare diseases. Means and standard deviations for the course evaluation items are shown in table 2. Regarding the organization of the course, 91% of the participants felt that the information about the seminar provided in advance was accurate. 86% felt that the objectives and content described in the announcement were largely or completely in line with what was delivered in the seminar. All agreed largely or completely that the online format was appropriate for addressing objectives and content. 94% of the participants reported that the design of the seminar largely or completely supported their active participation. Technical aspects were deemed satisfactory by all. 94% were motivated by their participation to take part in further training on the topic of illness and school. Figures 2 and 3 illustrate the frequencies for the organizational aspects and motivation to participate in further training.

**Table 2:** Course evaluation means and standard deviations on a six-point Likert Scale.


	M	SD

I enjoyed attending the course	5.30	1.22

I benefitted from the course for my work at school	5.05	1.43

I feel well prepared for future work with students with rare diseases	4.75	1.41


## Discussion

Offering this seminar as part of teacher training and professional development leads to greater awareness of rare diseases in schools and encourages teachers to address this issue in order to provide appropriate support for affected students. Studies by Rosselló et al. (2018) and Voigtländer et al. (2012) illustrate that medical successes can increase the life expectancy and quality of life of those affected, so that many children and adolescents can successfully attend school. Nevertheless, there are still hurdles in everyday life and at schools, which is why (future) teachers should be specifically educated and trained (Voigtländer et al. 2012). This online seminar is a small contribution for achieving this goal. The online format enables easy access from any location and thus offering an opportunity for broad implementation. The involvement of various professions in the school is also very important in order to ensure good support (Rosselló et al. 2018).

The actual study is limited to the first cohort/implementation of the online seminar. Consequently, the sample size is very small, limited in its representativeness and possibly consists of participants who were particularly motivated for the topic. Nevertheless, they had little prior knowledge and experience and even in the small sample, statistically significant effects could be achieved. Due to the online format, the participation of a larger number of teachers from different federal states will be possible in the future. Another limiting factor is that the measurements are based purely on self-assessments. Nevertheless, self-efficacy has already proven to be a good predictor of performance in many studies (e.g., in the meta-analysis by Stajkovic & Luthans 1998, r = .38) and the competence screening has also been validated in other domains (Bergsmann et al. 2017).

In the future, it would be desirable to use additional methods, such as interviews with former participants and their students or classroom observations, in order to obtain more ecologically valid data on how the acquired knowledge is applied in school. Currently, the online seminar is only available in German. An English version for wider applicability could be implemented.

## Conclusion

The seminar has established itself in teacher training and professional development and is already being offered at other University Colleges of Teacher Education in Austria. Additionally, the format has been successfully applied to other conditions such as diabetes, epilepsy, and mental or psychosomatic illnesses. Voigtländer et al. (2012) report an increased focus on rare diseases noting an improved overall and detailed knowledge base. To further advance this progress, educational efforts and professional development opportunities should be continued (Voigtländer et al. 2012).

The positive developments observed in the online seminar regarding self-efficacy and competency development support the expansion of the program to reach a broader population of current and future teachers. These individuals can integrate their knowledge into their respective schools and act as multipliers. Further, the instructional unit selected by Eurordis is an excellent example of how an online seminar can have global impacts and contribute to increasing understanding of affected children and adolescents, ultimately improving their situation.
